# Hæmoptysis Treated by Ergot

**Published:** 1876-09

**Authors:** 


Hæmoptysis Treated by Ergot—Report of Fifty Cases.—Dr. J. M. Williamson (*London Lancet,* January, 1876) reports fifty consecutive cases of hæmoptysis treated by ergot. Out of these the drug rapidly checked bleeding in forty-four cases. In the other six it failed, as did also gallic acid.—*Physician and Pharmacist.*

